# Case Report: Multidisciplinary discussion and management of a case involving a non-seminomatous germ cell tumor complicated by the presence of cancerous thrombus in the inferior vena cava

**DOI:** 10.3389/fonc.2025.1532983

**Published:** 2025-04-16

**Authors:** Qian Wang, Hong Zeng, Qiyu Zhu, Jindong Dai, Ming Zhang, Sicheng Wan, Pengfei Shen

**Affiliations:** Department of Urology, Institute of Urology, West China Hospital, Sichuan University, Chengdu, Sichuan, China

**Keywords:** non-seminomatous germ cell tumors, inferior vena cava, multi-disciplinary team, tumor thrombus, chemotherapy

## Abstract

Testicular cancer predominantly manifests in younger males. It demonstrates a favorable response to cisplatin-based chemotherapy and exhibits a promising overall survival. Nevertheless, the emergence of certain rare complications, such as the development of an embolus in the vena cava due to cancer, suggests that the tumor exhibits a high degree of aggressiveness and is associated with a poorer prognosis. We present a case of stage IIIC mixed malignant germinoma with metastases to both lungs, left kidney, right paravascular iliac lymph nodes, periaortic lymph nodes, and an inferior vena cava tumor thrombus. Following Multi-disciplinary Team (MDT) discussion, treatment modalities including surgery and adjuvant chemotherapy were devised, resulting in optimal therapeutic outcomes without complications or significant adverse reactions.

## Introduction

Testicular cancer represents 1% of adult tumors and 5% of urinary system tumors. At the time of initial diagnosis, germ cell tumors account for 90-95% of cases and 1-2% are bilateral (1). Invasion of the inferior vena cava (IVC) to form a cancerous thrombus is uncommon in non-seminomatous germ cell tumors (NSGCT), representing only 1% of metastatic disease in NSGCT, and is correlated with an increased risk of pulmonary embolism (2). In cases of IVC involvement, the primary tumor is typically located on the right side due to the more favorable anatomy of the right gonadal vasculature (3). In most cases, cancer thrombus in the inferior vena cava is identified through imaging studies and typically does not present with early symptoms (4, 5). As the condition progresses, it primarily results in bilateral lower limb edema (6). In patients with germ cell tumors complicated by inferior vena cava cancer thrombus, the primary tumor should be resected first to achieve debulking and obtain pathological confirmation. Management of the inferior vena cava cancer thrombus requires meticulous planning, often necessitating multidisciplinary evaluation, including vascular surgery, for optimal surgical intervention. Postoperatively, most patients typically opt for adjuvant chemotherapy to reduce the size of the cancer thrombus (4–7). Following appropriate therapeutic interventions, the 5-year survival rate exceeds 90%. For patients with advanced metastatic disease, this rate decreases to 70% (2). Notably, patients with inferior vena cava tumor thrombus are at an elevated risk of thrombus-related complications and distant metastasis (4).

The management of germ cell tumors involves a prolonged treatment duration and a sophisticated treatment regimen, necessitating multidisciplinary collaboration. Throughout the treatment process, a multidisciplinary team comprising surgeons, oncologists, and radiologists is required to conduct evaluations. Enhancing patient survival rates can be achieved through an integrated multidisciplinary collaborative approach. We present the case of a 31-year-old male diagnosed with right testicular NSGCT who was managed through a multidisciplinary approach. The patient had metastatic carcinoma with embolism affecting both lungs, the left kidney, retroperitoneal lymph nodes, and the inferior vena cava.

## Case presentation

A 31-year-old male, with a history of right testicular descent fixation for cryptorchidism, presented to the hospital with complaints of right testicular swelling and pain persisting for one month. Physical examination revealed notable enlargement of the right testicle, accompanied by mild erythema and tenderness of the scrotal skin. There were no associated lower urinary tract symptoms or other discomforts. Color Doppler ultrasound of the male reproductive system revealed a solid testicular mass on the right side. The abdominal enhanced computed tomography (CT) scan revealed a significant enlargement of the right testicle, measuring approximately 6.5cmx7.0cm in cross-section. Additionally, multiple enlarged lymph nodes were observed between the abdominal aorta and IVC, with the largest measuring about 1.5cm. The chest CT scan revealed multiple circular nodules in both lungs, with the largest measuring approximately 0.2-2.3cm, raising suspicion of metastatic tumors. The hematological examination revealed an alpha-fetoprotein (AFP) level of 3000ug/L, beta-human chorionic gonadotropin (B-HCG) level of 20.3IU/L, and lactate dehydrogenase (LDH) level of 802U/L, all other hematologic parameters were within normal range. After admission, the preoperative evaluation revealed no surgical contraindications, and he underwent right orchiectomy and right inguinal hernia repair. Intraoperatively, it was noted that the neck of the hernia sac protruded beyond the inferior epigastric artery with an inner ring diameter of approximately 2cm. A 3.0x5.0cm hernia sac with significant adhesions to surrounding tissues was identified, containing omentum majus, thickened spermatic cord, and an enlarged right testicle measuring approximately 7.0x9.0cm.

**Figure 1:**
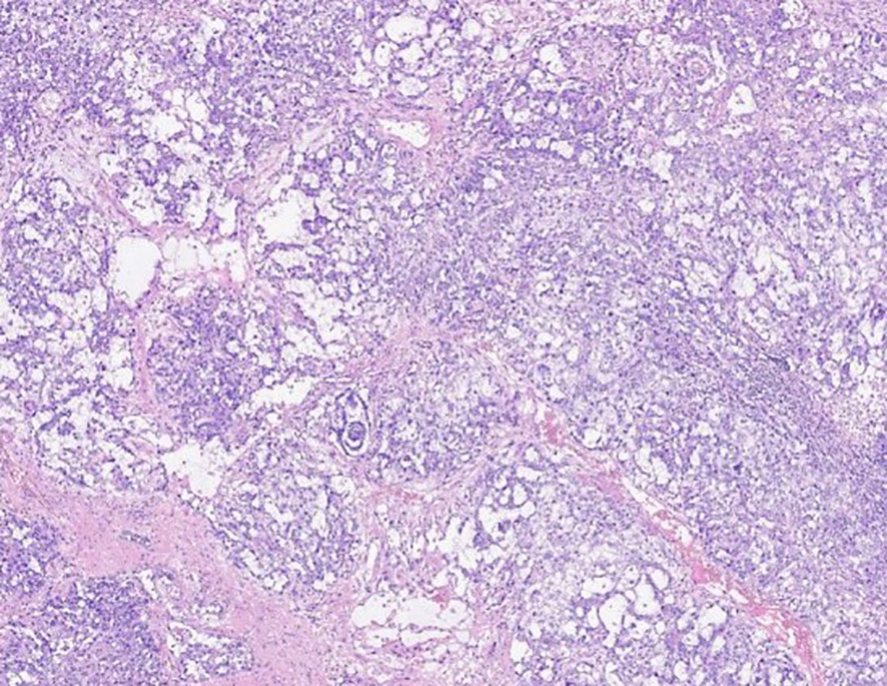
Hematoxylin and eosin staining of the right testicle reveals mixed malignant germinoma (mainly consisting of embryonal carcinoma and yolk sac tumor components).

The histological specimen was thoroughly examined by an experienced pathologist and diagnosed as mixed malignant germ cell tumor (mainly comprising embryonic carcinoma and yolk sac tumor components, with a small amount of suspected teratoma mucinous gland components) and the presence of vascular tumor embolus.The results of immunohistochemical pathology showed that the positive rate of SALL4(+), HCG (-), AFP (small focus +), CD117(-) and Ki-67 was approximately 80%. Components of embryonic cancer included: OCT3/4(+), PLAP (weak +), CD30 (+), GPC3(weak +); Yolk sac tumor components included: OCT3/4(-), GPC-3(+). Mucinous glands showed: PCK (+), CD30(-), GPC-3(-) (**Figure 1**).

**Figure 2:**
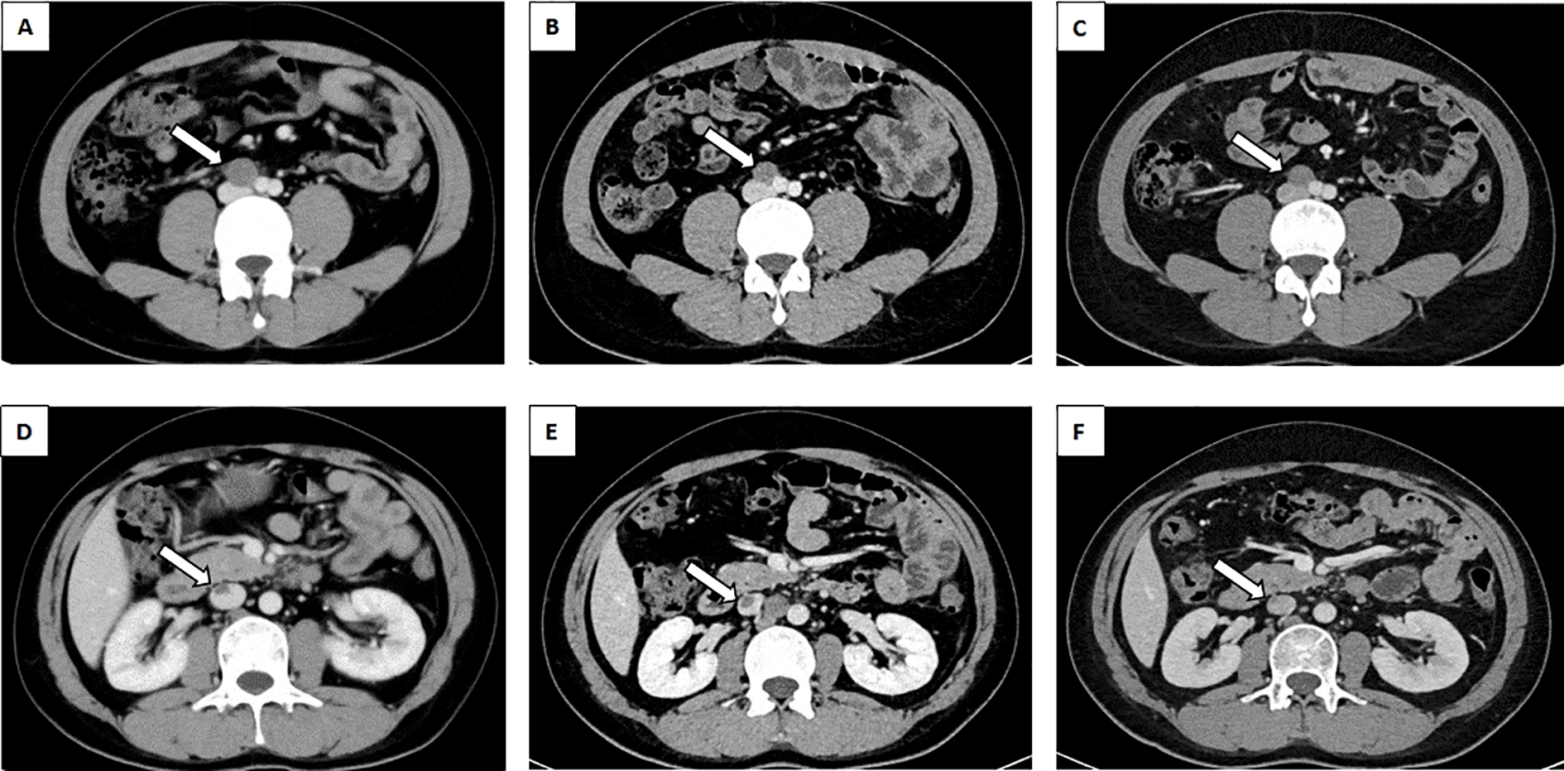
Abdominal CT revealed lymph nodes between the abdominal aorta and IVC. **(A)** lymph nodes prior to chemotherapy(white arrow). **(B)** The BEP regime for two cycles of chemotherapy for lymph nodes (white arrow). **(C)** The TIP regime for one cycle of chemotherapy for lymph nodes (white arrow). Abdominal CT revealed malignant thrombus of the IVC. **(D)** malignant thrombus of the IVC prior to chemotherapy(white arrow). **(E)** The BEP regime for two cycles of chemotherapy for malignant thrombus of the IVC (white arrow). **(F)** The TIP regime for one cycle of chemotherapy for malignant thrombus of the IVC (white arrow). CT, Computed Tomography; BEP, Bleomycin, Etoposide, Cisplatin; TIP, Paclitaxel, Ifosfamide, Cisplatin; IVC, Inferior vena cava.

**Figure 3:**
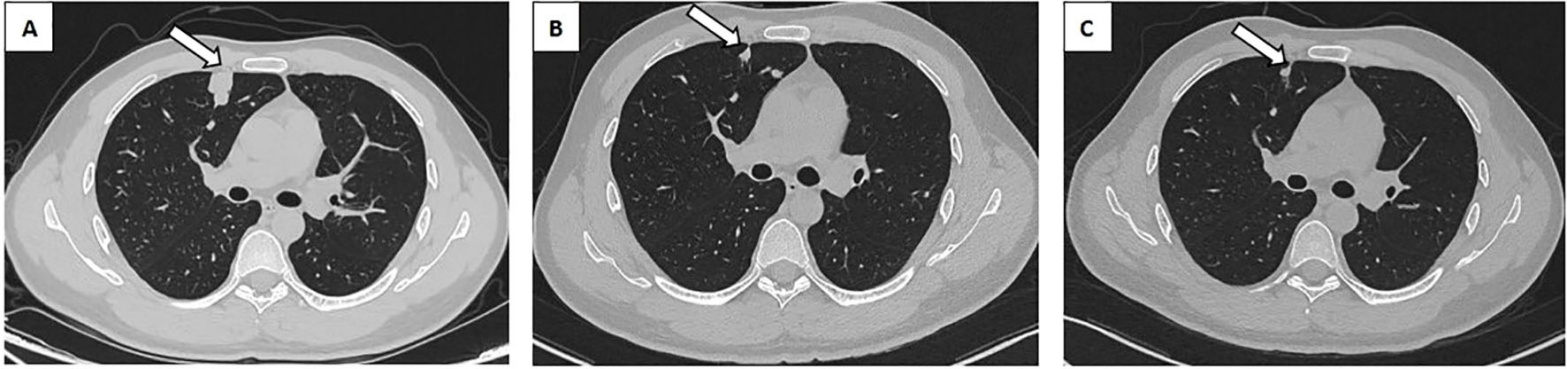
Chest CT revealed a metastatic tumor in the right lung. **(A)** Metastatic tumor of the right lung prior to chemotherapy (white arrow). **(B)** The BEP regime for two cycles of chemotherapy for right lung metastasis (white arrow). **(C)** The TIP regime for one cycle of chemotherapy for right lung metastasis (white arrow). CT, Computed Tomography; BEP, Bleomycin, Etoposide, Cisplatin; TIP, Paclitaxel, Ifosfamide, Cisplatin.

One month following the surgery, an enhanced CT scan of the patient’s abdomen demonstrated enlargement of the right circumiliac and periaortic lymph nodes, with partial fusion, invasion of the horizontal segment of the duodenum and the anterior portion of the left kidney, as well as invasion of the inferior vena cava resulting in a 1.1x3.8cm cancer thrombus(**Figures 2A, D**). Additionally, chest enhanced CT scan indicated multiple nodules in both lungs measuring approximately 0.2-2.3cm, suggestive of metastatic tumors (**Figure 3A**). The hematological examination revealed an AFP level of 910ug/L, B-HCG level of 4.58IU/L, and LDH level of 320U/L. The final diagnosis was a high risk stage IIIC pT3N2M1bS2 right testicular mixed malignant germ-cell tumor, predominantly composed of embryonic carcinoma and vitellinoma components, with metastasis to both lungs, left kidney, right paravascular iliac lymph nodes, periaortic abdominal lymph nodes, and the formation of cancerous thrombus in the inferior vena cava. After conducting a Multi-disciplinary Team (MDT) discussion, the BEP regimen (bleomycin, etoposide, cisplatin) was chosen for adjuvant chemotherapy in consideration of the patient’s current multiple metastases throughout the body and in accordance with clinical guidelines (8). Due to the presence of an inferior vena cava cancer embolus extending to the level of the renal vein, vascular surgeons deemed it inappropriate to proceed with installation of an inferior vena cava filter, opting instead for anticoagulant therapy to monitor changes in the cancer embolus.

After 2 cycles of BEP chemotherapy, the reexamination CT scan revealed that the cancer thrombus in the inferior vein had enlarged(**Figures 2B, E**), the pulmonary nodules had grown larger(**Figure 3B**), AFP was 4.64 ug/L, B-HCG was 12.86 IU/L and LDH was 214U/L, the comprehensive efficacy was assessed as progress disease (PD), and the patient presented with myelosuppression (leukocyte 1.29 x 10^9^). After the MDT discussion, it was determined that the chemotherapy regimen would be changed to the TIP regimen (paclitaxel, ifosfamide, cisplatin) for 2 cycles.

**Figure 4:**
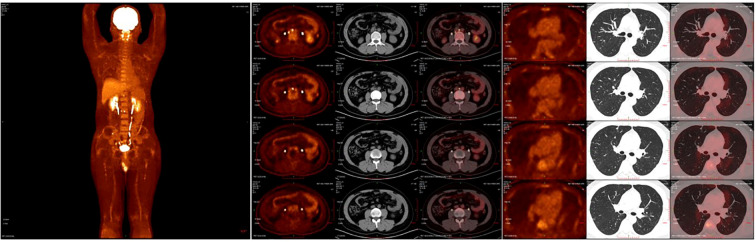
**(A)** The PEC-CT scan reveals the systemic tumor burden after 4 cycles of chemotherapy. **(B)** The PET-CT scanning revealed that glucose metabolism was elevated in the vicinity of the right external iliac vessel and the abdominal para-aortic lymph nodes. **(C)** The PET-CT scan showed no lesion of increased glucose metabolism in both lungs. PET-CT, Positron Emission Tomography - Computed Tomography.

After the completion of the first cycle of TIP chemotherapy, the CT scan indicated that the right para-iliac and periaortic lymph nodes were diminished and contracted (**Figure 2C**), the cancer thrombus in the inferior vena cava was remarkably reduced (**Figure 2F**), the metastatic tumors in both lungs were decreased (**Figure 3C**), AFP was 3.58 ug/L, HCG was 0.6 IU/L and the comprehensive clinical efficacy evaluation was a partial response (PR). After the conclusion of the second cycle of TIP chemotherapy, Positron Emission Tomography - Computed Tomography (PET-CT) scanning revealed that glucose metabolism was elevated in the vicinity of the right external iliac vessel and the abdominal para-aortic lymph nodes, suggesting tumor metastasis with a size of 0.7-0.8cm (**Figure 4**). The comprehensive efficacy evaluation was PR. After the MDT discussion, it was determined that the subsequent step would be the surgical removal of the residual lesion to achieve the optimal therapeutic effect.

## Discussion

The incidence of NSGCT and mixed germ cell tumors peak around 30 years of age, while the development of inferior vena cava cancer thrombus is exceedingly rare (9, 10). Retroperitoneal lymph node metastasis frequently occurs in patients with inferior vena cava thrombus formation (4). Kidney metastases are exceedingly uncommon across all cancer types, not limited to testicular cancer (11). After four cycles of chemotherapy, there was a significant reduction in the size of the thrombus and abdominal metastases associated with inferior vena cava cancer. A recent PET-CT scan revealed no evidence of residual thrombus or disease activity in the inferior vena cava. To optimize the treatment outcome, we have decided to proceed with surgical resection of any remaining lesions.

Donohue et al. (12) reported that among 530 patients who underwent retroperitoneal residual mass resection after chemotherapy, 42 (7%) had experienced inferior vena cava wall invasion. Husband et al. (2) observed that 4 out of 397 patients with metastatic testicular GCT exhibited involvement of the inferior vena cava. According to the Beck report, 72.7 percent of patients with metastatic spread to the IVC had a primary tumor in the right testicle (10). The mechanism of metastatic testicular carcinoma thrombus invasion of the inferior vena cava (IVC) may result from direct invasion of the spermatic vein and subsequent drainage into the vena cava. This pathway provides further insight into the right testicular tumor’s invasion of the IVC, as the right gonadal vein directly connects to the IVC. The mechanism of metastatic testicular carcinoma thrombus invasion of the inferior vena cava (IVC) may result from direct invasion of the spermatic vein and subsequent drainage into the vena cava. The alternative mechanism involves the spread of lymph to the paraval or intercaval lymph nodes, leading to a direct invasion of the IVC through a lymphatic-venous shunt. Therefore, giant retroperitoneal disease can be considered a risk factor for IVC cancer metastasis, which may be applicable in our patient’s case and could account for the presence of cancer metastases in the IVC (12, 13).

The most concerning complication of IVC thrombosis is pulmonary embolism (PE), which can result in sudden death (13). Bredael et al. (14) reported a 9% mortality rate attributed to PE in a cohort of patients with advanced testicular cancer. Furthermore, despite the administration of anticoagulant therapy, the risk of PE persists. During chemotherapy, tumor necrosis resulting from the treatment may lead to its migration and subsequently result in pulmonary embolism (15). This finding prompted consideration of IVC filter placement prior to treatment initiation as a preventive measure against PE occurrence. After an MDT discussion regarding the patient, it was determined by the vascular surgeon that the cancer thrombus had extended to the level of the renal vein, rendering it unsuitable for IVC filter placement. Consequently, anticoagulant therapy was initiated and close monitoring of changes in the cancer thrombus ensued. Therefore, for testicular cancer patients with inferior vena cava tumor thrombus, inferior vena cava filters should be implanted or only anticoagulant therapy should be administered according to the location of the tumor thrombus.

Although vena cava involvement in NSGCT is relatively rare, it is more frequently observed in other types of cancer, such as renal (16) and hepatocellular carcinoma (17). However, there is no universally accepted standard for the optimal treatment approach. The therapeutic strategy for cancer involving the vena cava varies depending on specific clinical scenarios. The management of IVC cancer thrombus primarily involves surgical resection, with various surgical techniques available. Kinebuchi et al. successfully excised an inferior vena cava cancer thrombus using veno-venous bypass (18). Paule et al. (19) documented a case of testicular embryonic carcinoma with tumor embolus extending to the IVC and right atrium, in which intravascular and intracardiac neoplastic masses were successfully excised utilizing cardiopulmonary bypass. Zhang et al. presented a case of robot-assisted laparoscopic resection for testicular cancer with an IVC tumor thrombus (7). Chemotherapy alone can also be utilized for the eradication of IVC cancer thrombus, albeit occurrences of this nature are exceedingly rare (20). Furthermore, long-term follow-up monitoring plays a crucial role in disease management. The primary objective of long-term follow-up is the early detection of recurrent or metastatic lesions and the ongoing assessment of long-term chemoradiotherapy toxicity. AFP and HCG serve as significant biomarkers, with approximately two-thirds of patients experiencing recurrent non-seminomatous germ cell tumors exhibiting elevated marker levels. Abdominal and pelvic CT remains the principal imaging modality (21). Lymphatic invasion, tumor composition, and staging (particularly a high proportion of embryonal carcinoma) are key risk factors for recurrence (9, 22). In our case, the cancer thrombus in the IVC was completely eradicated following TIP regimen chemotherapy. Concurrently, considering reported instances of chemotherapy-related deep vein thrombosis complications (23), we also administered anticoagulation therapy and conducted dynamic monitoring of coagulation function along with lower limb venous color ultrasound. Therefore, patients diagnosed with testicular cancer and IVC cancer thrombus should undergo surgical evaluation for resection either before or after chemotherapy. Irrespective of the decision to proceed with surgery, all patients require appropriate anticoagulant therapy to ensure safety.

## Conclusion

The formation of IVC thrombus is an uncommon complication associated with testicular cancer and should be thoroughly considered in the diagnostic process. In the setting of malignancy, prompt and accurate evaluation, along with appropriate treatment, is crucial to minimize the risk of potentially life-threatening pulmonary embolism.

## Data Availability

The raw data supporting the conclusions of this article will be made available by the authors, without undue reservation.
